# Maternal Obesity Alters Neurotrophin-Associated MAPK Signaling in the Hypothalamus of Male Mouse Offspring

**DOI:** 10.3389/fnins.2019.00962

**Published:** 2019-09-13

**Authors:** Inga Bae-Gartz, Ruth Janoschek, Saida Breuer, Lisa Schmitz, Thorben Hoffmann, Nina Ferrari, Lena Branik, Andre Oberthuer, Cora-Sophia Kloppe, Sarah Appel, Christina Vohlen, Jörg Dötsch, Eva Hucklenbruch-Rother

**Affiliations:** ^1^Department of Pediatrics, University Hospital of Cologne, Cologne, Germany; ^2^Heart Center, Cologne Center for Prevention in Childhood and Youth, University Hospital of Cologne, Cologne, Germany

**Keywords:** synaptic plasticity, BDNF, HFD, microarray, neurogenesis, CAMKII phosphorylation, IBA1, vGLUT2

## Abstract

**Purpose:**

Maternal obesity has emerged as an important risk factor for the development of metabolic disorders in the offspring. The hypothalamus as the center of energy homeostasis regulation is known to function based on complex neuronal networks that evolve during fetal and early postnatal development and maintain their plasticity into adulthood. Development of hypothalamic feeding networks and their functional plasticity can be modulated by various metabolic cues, especially in early stages of development. Here, we aimed at determining the underlying molecular mechanisms that contribute to disturbed hypothalamic network formation in offspring of obese mouse dams.

**Methods:**

Female mice were fed either a control diet (CO) or a high-fat diet (HFD) after weaning until mating and during pregnancy and gestation. Male offspring was sacrificed at postnatal day (P) 21. The hypothalamus was subjected to gene array analysis, quantitative PCR and western blot analysis.

**Results:**

P21 HFD offspring displayed increased body weight, circulating insulin levels, and strongly increased activation of the hypothalamic insulin signaling cascade with a concomitant increase in ionized calcium binding adapter molecule 1 (IBA1) expression. At the same time, the global gene expression profile in CO and HFD offspring differed significantly. More specifically, manifest influences on several key pathways of hypothalamic neurogenesis, axogenesis, and regulation of synaptic transmission and plasticity were detectable. Target gene expression analysis revealed significantly decreased mRNA expression of several neurotrophic factors and co-factors and their receptors, accompanied by decreased activation of their respective intracellular signal transduction.

**Conclusion:**

Taken together, these results suggest a potential role for disturbed neurotrophin signaling and thus impaired neurogenesis, axogenesis, and synaptic plasticity in the pathogenesis of the offspring’s hypothalamic feeding network dysfunction due to maternal obesity.

## Introduction

The rising incidence of maternal obesity is a worldwide phenomenon and the relationship between maternal obesity and the offspring’s long-term predisposition for metabolic disorders, such as obesity and type-2-diabetes, is undeniable (Howie et al., 2009; Plagemann, 2011; O’Reilly and Reynolds, 2013). Human and rodent studies have led to the description of novel interactive pathways between the maternal environment and the fetus (Poston, 2012). In particular, there is a clear link between maternal adiposity and expression and function of important modulators of energy homeostasis in adipose tissue, liver and the brain of the offspring (Bouret, 2009; Catalano et al., 2009; McCurdy et al., 2009; Li et al., 2011; Maric-Bilkan et al., 2011; Rooney and Ozanne, 2011; Zhang et al., 2011).

The central control of energy homeostasis is a complex process regulated by a collection of neurons primarily located in the mediobasal hypothalamus. Specifically, the melanocortin system of the arcuate nucleus of the hypothalamus (ARC) has been shown to convey anorexigenic and orexigenic signals in response to circulating hormones and nutrients (Elias et al., 1999; Cowley et al., 2001; Aponte et al., 2011; Krashes et al., 2011). While early studies in rodents focused on analyzing the effects of hormones or nutrients on anorexigenic or orexigenic neuropeptide gene expression in the ARC, research interest shifted toward a deeper functional understanding of acute effects of substances like insulin, leptin, ghrelin, or glucose on electrophysiological properties of neuropeptide releasing ARC producing cells (Cowley et al., 2001, 2003a,b; Jobst et al., 2004; Pinto et al., 2004; van den Top et al., 2004; Dhillon et al., 2006; Konner et al., 2007). A number of studies described plasticity of synaptic input patterns as an important component of such changes in neuronal activity (Horvath and Diano, 2004; Pinto et al., 2004; Horvath and Gao, 2005; Sternson et al., 2005; Gao et al., 2007; Benani et al., 2012). For instance, leptin was shown to have a major influence on synaptic organization of orexin neurons in the lateral hypothalamus (Horvath and Gao, 2005), whereas ghrelin was found to impact synapse formation in various extra-hypothalamic brain regions (Abizaid et al., 2006; Diano et al., 2006). Furthermore, high-fat feeding in rodents was also linked to changes in the synaptic input pattern of hypothalamic feeding neurons (Benani et al., 2012), and it was found that rats prone to diet-induced obesity display a completely different pattern of synaptic inputs in the ARC compared to diet-resistant littermates before even being exposed to high-fat feeding (Horvath et al., 2010). In conclusion, there is accumulating evidence that synaptic plasticity induced by metabolic cues plays a pivotal role in determining the function of hypothalamic neuronal circuits regulating appetite.

Neurotrophins are a family of ubiquitously expressed growth factors controlling development, survival, and function of neurons (Hempstead, 2006; Reichardt, 2006) that consist of six members: nerve growth factor (NGF), brain-derived neurotrophic factor (BDNF), neurotrophin 3 (NT3), neurotrophin 4/5 (NT4/5), ciliary neurotrophic factor (CNTF), and the glia cell derived neurotrophic factor (GDNF) group of ligands. Upon secretion, which is usually triggered by membrane depolarization (Blochl and Thoenen, 1996; Lessmann et al., 2003), they are capable of mediating many activity-dependent central processes, including neuronal differentiation and growth, synapse formation and plasticity of synaptic input patterns (Park and Poo, 2013). There are two distinct classes of neurotrophin receptors (NTR) that can bind to neurotrophins: p75NTR, which binds to all neurotrophins, and various subtypes of tropomyosin receptor kinase (Trk) receptors, which are each specific for different neurotrophins. Among the neurotrophins, BDNF has gained much attention in the context of energy homeostasis regulation – not for its well described positive effects on synaptogenesis and neuronal plasticity (Unger et al., 2007), but its anorexigenic effect (Rask-Andersen et al., 2011). In humans, a polymorphism in the BDNF gene has been linked to obesity (Hotta et al., 2009; Thorleifsson et al., 2009). Furthermore, BDNF-deficient mice exhibit obesity due to overfeeding (Kernie et al., 2000), a phenotype that is mirrored in mice deficient for the high-affinity BDNF receptor TrkB (Xu et al., 2003). However, to what extent BDNF’s anorexigenic effects are mediated by its action on synaptic plasticity or neuronal differentiation remains to be determined. Given the fact that an increasing number of studies indicate that synaptic plasticity in the hypothalamus plays a crucial role in the control of energy homeostasis (Pinto et al., 2004; Yang et al., 2011; Liu et al., 2012), it is very likely that BDNF and other less well characterized neurotrophins might influence hypothalamic feeding network formation and function (Vanevski and Xu, 2013).

In the context of maternal obesity and the offspring’s predisposition to obesity and diabetes in later life, the hypothalamus has emerged as a particularly vulnerable organ (Bouret, 2009). Hypothalamic dysfunction in the offspring of overfed or obese mothers has been suggested by several animal studies, predominantly showing the effect of maternal adiposity and diet on hypothalamic neuropeptide and hormone receptor expression or, more recently, reactive inflammation-associated hypothalamic neuronal dysfunction (Cottrell and Ozanne, 2007; Cottrell et al., 2009; Alfaradhi and Ozanne, 2011; Poston, 2012; Rother et al., 2012; Velloso, 2012). Furthermore, a number of studies suggested stable wiring of hypothalamic feeding networks as an important pathomechanism in hypothalamic dysfunction resulting from increased circulating hormone levels in maternal obesity (Bouret et al., 2004a, b; Pinto et al., 2004; Bouret and Simerly, 2006; Bouret, 2009). Especially, insulin and leptin have been shown to permanently change hypothalamic network formation during the critical time window of perinatal development (Bouret and Simerly, 2006; Ozanne, 2011). However, the role of neurotrophins as a potential link between a metabolically disturbed intrauterine milieu and altered formation of hypothalamic neuronal networks responsible for the regulation of feeding behavior and energy balance has not been addressed to date.

## Materials and Methods

### Animal Care

All animal procedures were conducted in compliance with protocols approved by the Committee on the Ethics of Animal Experiments of the Landesamt für Natur, Umwelt und Verbraucherschutz Nordrhein-Westfalen (Permit number: 37.09.292, RDA number: 84-02.04.2014.A057) and were in accordance with National Institutes of Health guidelines. Mice (C57BL/6N) were bred locally at a designated animal unit of the University Hospital of Cologne (Cologne, Germany). All animals were housed individually and were maintained at 22°C on a 12 h light, 12 h dark cycle. As bedding, spruce granulate (Lignocel FS 14; Rettenmaier & Söhne GmbH, Germany) was provided. Nestlets, mouse smart home and aspen bricks served as enrichment (Plexx B.V., The Netherlands). Animal care and use was performed by qualified individuals and supervised by a veterinarian. The manuscript complies with the Animals in Research: Reporting *in vivo* Experiments (ARRIVE) guidelines (Kilkenny et al., 2010).

Three weeks old female mice were fed a standard chow (#.R/M-H SSniff, Soest containing 41.2% carbohydrates, 19% protein, and 3.3% fat; 9% of calories from fat) or high-fat diet (#C1057 modified containing 26.9% carbohydrates, 21% protein, and 35.1% fat; 60% of calories from fat) for 9–10 weeks preconception and during gestation and lactation, yielding sedentary control (CO; *n* = 20) and high-fat diet (HFD; *n* = 21) group. Male breeders were standard chow-fed and had access to the HFD during mating process (48 h). Male breeders that were in contact with HFD for two times were excluded for further mating. Water and food were available *ad libitum*. Body weight of the dams was monitored daily from G0 to G18. Blood samples for serum analyses were collected 1 week before mating (named G0) and at G15. In order to minimize stress-induced side effects, dams and offspring were not fasted and were collected between 9 and 10o’clock in the morning. After delivery, body weight of the pups was monitored daily starting within 24 h after birth. On P2, litter size was adjusted to six for each litter. Smaller litters were excluded from the experiment (CO *n* = 2, HFD *n* = 2). On P21, male pups were sacrificed non-fasted and blood samples were collected via intracardial puncture for further analyses. Organs were excised. The hypothalamus was cut immediately caudal to the optic chiasm. The dissection was limited laterally by the hypothalamic sulci and dorsally by the mammillothalamic tract (Rother et al., 2012). The remaining brain tissue was also preserved, both epigonadal fat pads were harvested, the weight was determined and all tissues were immediately frozen at −80°C for further analysis. A maximum of two offspring per dam were analyzed to minimize litter-dependent bias. All studies were performed using male offspring. Cohorts used for this study represent a subset of animals that were also used in another study by our group, see Bae-Gartz et al. (2016).

### Analytical Procedures

Serum levels of insulin and leptin were measured by ELISA using mouse standards according to the manufacturer’s guidelines (mouse insulin ELISA (EZRMI-13K and EZML-82K) with a sensitivity of 0.01 ng/ml and intra-assay variation of 1.06%; Millipore CorpBillerica, MA, United States).

### Intraperitoneal Glucose Tolerance Test

Glucose tolerance tests (GTT) were performed as previously described (Bae-Gartz et al., 2016). Briefly, animals were fasted for 16 h (18:00–10:00 h). After determination of fasted blood glucose levels, each animal received an intraperitoneal (ip) injection of 20% glucose (10 ml/kg body weight = 2 g glucose/kg body weight). Blood glucose levels were measured after 15, 30, 60, and 120 min.

### Quantitative PCR

Total RNA was isolated from hypothalamus of CO and HFD animals using TRI-Reagent^®^ (Sigma-Aldrich) according to the manufacturer’s guidelines. RNA quantity and purity were determined by measuring UV absorption with a Tecan spectrophotometer (Tecan, Nano Quant infinite M200 Pro). Quantitative changes in mRNA expression for genes encoding *Bdnf, Ngf, Nt3, Nt4/5, Cntf, Gdnf, TrkA, TrkB, TrkC, p75Ntr, Prr7, Arc, Egr, Syt1, Homer* were assessed by quantitative real-time PCR as described previously using the 7500 Real-time PCR system (Applied Biosystem, Foster City, CA, United States) or the IQ TM SYBR-Green© Supermix and a BioRad iQ5-Cycler (Bio-Rad Laboratories, Hercules, CA, United States) (Plank et al., 2006; Rother et al., 2012). In all samples, the relative amount of specific mRNA was normalized to at least four ubiquitously expressed housekeeping genes (β-Actin, hypoxanthin-guanin-phosphoribosyltransferase (HPRT), glycerinaldehyd-3-phosphat-dehydrogenase (GAPDH), and β-glucuronidase (GUSB). Oligonucleotides were designed with Primer Express software (Perkin-Elmer, Foster City, CA, United States). Primer pairs and Taqman probes are listed in Supplementary Table 1.

### Microarray Analyses of the Hypothalamus

Microarray experiments of mice hypothalamic RNA were performed as single-color experiments using 4_44K Mouse (v2) Whole Genome Arrays from Agilent Technologies (Santa Clara, CA, United States) as described before (Rother et al., 2012). First, differentially expressed genes between the CO group and HFD group, were identified using the Rank Product test (Breitling et al., 2004). Genes were called significant with a percentage false positive below 0.001. All statistical calculations were performed using R version 3.1.2 and Bioconductor. Functional Annotation Clustering was performed by DAVID Bioinformatics Resources^1^.

The data discussed in this publication have been deposited in NCBI’s Gene Expression Omnibus (Edgar et al., 2002) and are accessible through GEO Series accession number GSE135830^2^.

### Western Blot Analysis

Frozen dissected hypothalamic tissue of CO and HFD offspring was homogenized in lysis buffer as previously described (Alejandre-Alcazar et al., 2007). Protein concentration was determined with a BCA-Protein Assay Kit (Thermo Fisher Scientific, Waltham, MA, United States). Lysates resolved on a 10% reducing SDS-PAGE gel were transferred to a nitrocellulose membrane. Blots were probed with the following antibodies, see Supplementary Table 2. Monoclonal mouse anti-mouse-β-Actin (Cell Signaling, # 3700, 1:1000) and anti-mouse GAPDH (Cell Signaling, # 2118, 1:1000) served as a loading control. Anti-mouse IgG, horseradish peroxidase (HRP)-linked (Cell Signaling, # 7076, 1:2000), and anti-rabbit IgG, HRP-linked (Cell Signaling, # 7074, 1:2000) were used as secondary antibodies.

### Statistical Analysis

Values are shown as means ± standard error of the mean (SEM). The results of realtime RT-PCR were calculated based on the ΔΔCt– method and expressed as fold induction of mRNA expression compared to the corresponding control group (1.0-fold induction). Densitometric analysis of protein bands was performed using Bio-Rad ImageLab software (Bio-Rad, Munich, Germany). Two tailed Mann-Whitney-test was used to test significance of differences between HFD and CO animals at given time points. A *p*-value less than 0.05 was considered significant. The calculations were performed according to a previous agreement with the Institute of Medical Statistics, Informatics and Epidemiology, University of Cologne.

## Results

### Metabolic Phenotype

**FIGURE 1:**
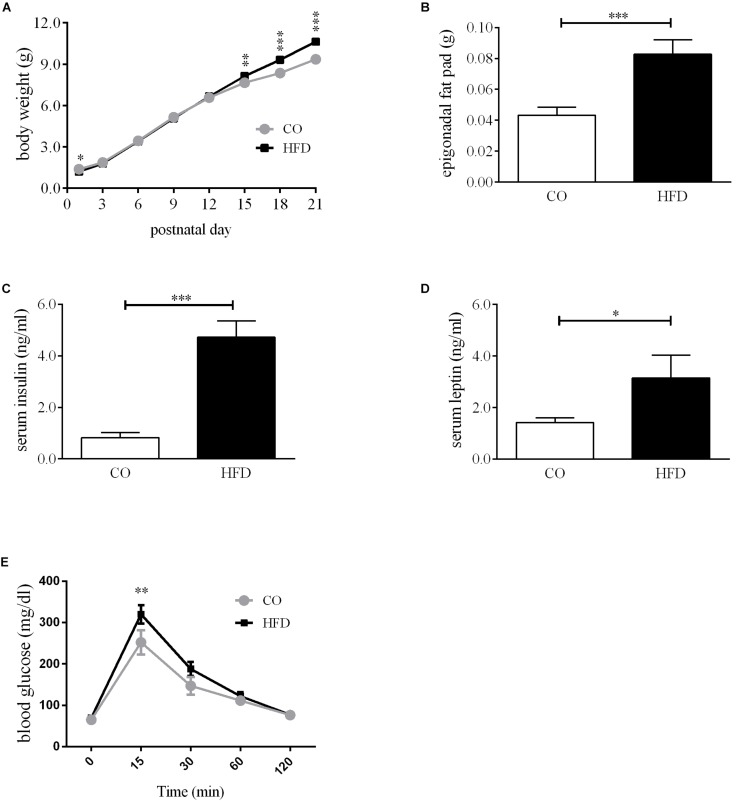
Offspring metabolic phenotype at P21. **(A)** Bodyweight gain offspring, *n* = 49–73. **(B)** Epigonadal fat pad weight, CO *n* = 35, HFD *n* = 22. **(C)** Serum insulin levels at P21, CO *n* = 8, HFD *n* = 6. **(D)** Serum leptin levels at P21, CO *n* = 7, HFD *n* = 6. **(E)** Intraperitoneal glucose tolerance test (GTT) at P21, CO *n* = 8 HFD *n* = 7. Data are presented as mean ± SEM. ^∗^*p* < 0.05; ^∗∗^*p* < 0.01; ^∗∗∗^*p* < 0.001; CO, control; HFD, high fat diet; g, gram; p, postnatal day.

To assess the metabolic consequences of maternal diet-induced obesity in the offspring, we first determined body weight gain and non-fasted insulin and leptin levels of the dams. Before mating, females on HFD were markedly heavier than controls (Supplementary Figure 1A). Maternal diet affected the body weight gain of the dams but at G18 the absolute body weight was similar in both groups (Supplementary Figure 1A). Serum insulin levels did not differ on a significant level between CO and HFD dams, but there was a strong insulin increase between G0 and G15 in HFD dams (Supplementary Figure 1B). Serum leptin levels revealed a marked increase in HFD dams at G0 and G15 (Supplementary Figure 1C). Litter size and sex ratio did not differ between groups (Supplementary Figures 1D,E). Portions of dams and offspring characteristics of this cohort of mice have been reported previously (Bae-Gartz et al., 2016; Schmitz et al., 2018). Offspring of obese mouse dams displayed significantly increased body weight and epigonadal fat pad weight at P21 (Figures 1A,B). Interestingly, HFD offspring had lower body weights at P1 (Figure 1A). Additionally, HFD offspring showed significantly increased non-fasted serum insulin and leptin concentrations at P21 compared to controls (Figures 1C,D). To assess the effects of maternal obesity to the offspring’s glucose metabolism, GTTs were performed. HFD offspring showed increased blood glucose levels compared with CO offspring 15 min after glucose injection in the intraperitoneal GTT (Figure 1E). At all other time points, there were no significant differences detectable.

### Hypothalamic Phenotype

**FIGURE 2:**
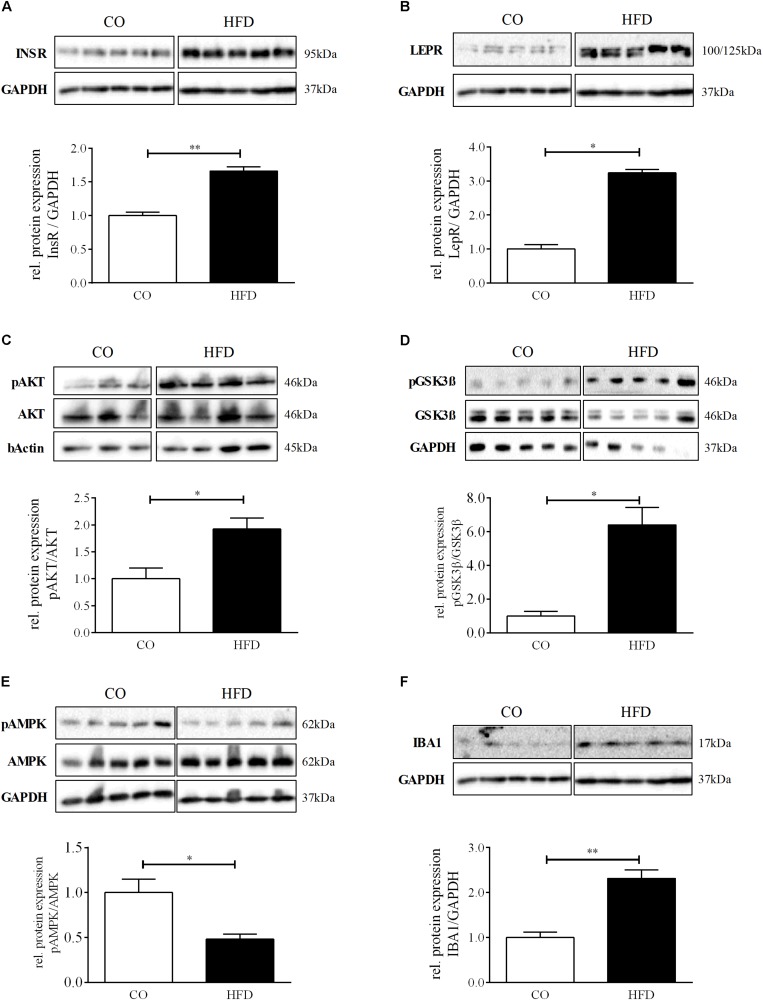
Hypothalamic phenotype at P21. **(A–F)** Representative Western Blots of INSR, LEPR, pAKT, pGSK3β, pAMPK, and IBA1 in hypothalamic tissue at P21. **(A–F)** Densitometric analysis of protein expression at P21: **(A)** INSR/GAPDH, CO *n* = 5, HFD *n* = 5. **(B)** LEPR/GAPDH, CO *n* = 5, HFD *n* = 5. **(C)** pAKT/AKT, CO *n* = 4, HFD *n* = 4. **(D)** pGSK3β/GSK3β, CO *n* = 5, HFD *n* = 4. **(E)** pAMPK/AMPK. **(F)** IBA1/GAPDH, CO *n* = 5, HFD *n* = 5. Data are presented as mean ± SEM. ^∗^*p* < 0.05; ^∗∗^*p* < 0.01; CO, control; HFD, high fat diet.

To examine whether maternal obesity also affects hypothalamic insulin and leptin signal transduction, we first quantified hypothalamic insulin and leptin receptor expression. Both, insulin and leptin receptor levels were significantly increased in HFD offspring at P21 (Figures 2A,B). Furthermore, we found significantly increased levels of phosphorylated RAC-alpha serine/threonine-protein kinase (pAKT) as well as increased protein levels of glycogen synthase kinase 3ß (pGSK3ß) in HFD offspring indicating robust activation of hypothalamic PI3K signaling at P21 following maternal obesity (Figures 2C,D). Consequently, hypothalamic 5′-Adenosine monophosphate-activated protein kinase (AMPK) phosphorylation was significantly decreased in HFD offspring (Figure 2E).

Additionally, we assessed hypothalamic protein levels of Ionized calcium-binding adapter molecule 1 (IBA1), a marker for microglial activation and promotion of reactive gliosis, that has been associated with diet-induced obesity and hypothalamic dysfunction in mice with a just recently described role in enhancing orexigenic AgRP expression upon insulin action (Valdearcos et al., 2017; Winkler et al., 2019). Interestingly, hypothalamic IBA1 protein levels were significantly increased in HFD offspring at P21 (Figure 2F). POMC and NPY mRNA levels revealed no significant difference between CO and HFD offspring (Supplementary Figure 2).

### Hypothalamic Transcriptome Analysis

**FIGURE 3:**
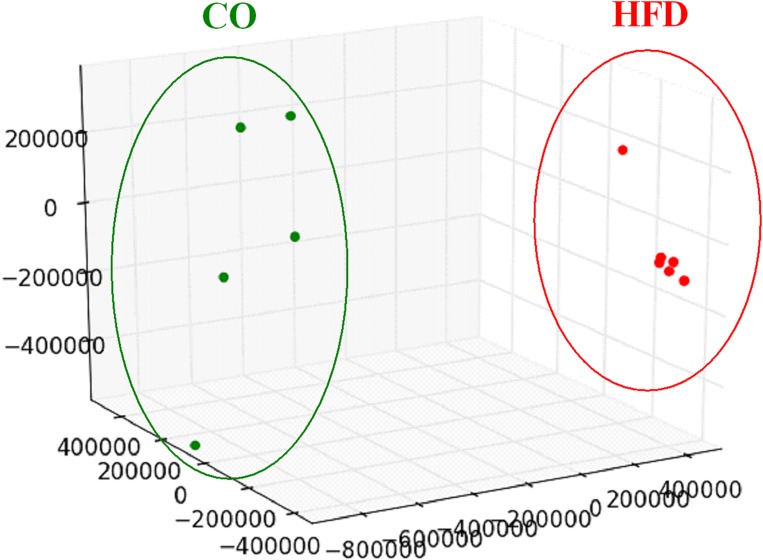
Hypothalamic transcriptome analysis at P21. Three-dimensional view of a principal component analysis of hypothalamic tissue performed with global gene expression information on male CO (*green dots*) and HFD (*red dots*) offspring at P21.

To evaluate the global hypothalamic gene expression profile accompanying the observed increase in insulin and leptin signaling and microglial activation at P21, we next performed genome-wide gene expression arrays with hypothalamic tissue of CO and HFD offspring (CO *n* = 5, HFD *n* = 6). Unsupervised principal component analysis revealed that global gene expression information differed markedly between CO and HFD offspring (Figure 3). To determine the affected biological processes, we next performed Functional Annotation Clustering of the differentially expressed genes via DAVID analysis (Supplementary Tables 3, 4, gene order and probes for differentially expressed genes of hypothalamic tissue at P21 upregulated and downregulated). Interestingly, numerous GO-Terms and KEGG-Pathways related to the field of synaptic plasticity and neuron development ranged among the most prominently enriched functionally related gene groups when comparing HFD and CO offspring (Table 1).

**TABLE 1:** Functional Annotation Clustering for differentially expressed genes between CO and HFD offspring.

**Category**	**Term**	***P*-value**	**FE**
KEGG_PATHWAY	mmu04080:Neuroactive ligand-receptor interaction	0.002	2,323
GOTERM_CC_FAT	GO:0045211∼postsynaptic membrane	0.005	2,873
GOTERM_CC_FAT	GO:0045211∼postsynaptic membrane	0.005	2,873
GOTERM_CC_FAT	GO:0032589∼neuron projection membrane	0.017	14,102
GOTERM_CC_FAT	GO:0031252∼cell leading edge	0.020	2,644
GOTERM_BP_FAT	GO:0048812∼neuron projection morphogenesis	0.022	2,095
GOTERM_BP_FAT	GO:0048858∼cell projection morphogenesis	0.026	1,966
GOTERM_CC_FAT	GO:0044456∼synapse part	0.028	2,018
GOTERM_CC_FAT	GO:0030054∼cell junction	0.029	1,610
GOTERM_BP_FAT	GO:0048666∼neuron development	0.029	1,749
GOTERM_BP_FAT	GO:0060080∼regulation of inhib. postsynaptic membrane potential	0.030	10,638
SP_PIR_KEYWORDS	postsynaptic cell membrane	0.032	2,439
GOTERM_BP_FAT	GO:0032990∼cell part morphogenesis	0.037	1,873
INTERPRO	IPR006028:Gamma-aminobutyric acid A receptor	0.040	5,183
GOTERM_CC_FAT	GO:0045202∼synapse	0.042	1,650
GOTERM_CC_FAT	GO:0031256∼leading edge membrane	0.042	8,974
GOTERM_CC_FAT	GO:0042995∼cell projection	0.044	1,488
GOTERM_BP_FAT	GO:0060081∼membrane hyperpolarization	0.046	8,510
GOTERM_BP_FAT	GO:0007409∼axonogenesis	0.048	1,914

*Enriched GO-Terms, Interpro and KEGG Pathways after Functional Annotation Clustering by DAVID Analysis. To simplify representation, only key nodes of the GO biological process tree found are shown. CO n = 5, HFD n = 6. GO, Gene Ontology; FE, fold enrichement; BP, Biological Process.*

### Hypothalamic Neurotrophin Expression

**FIGURE 4:**
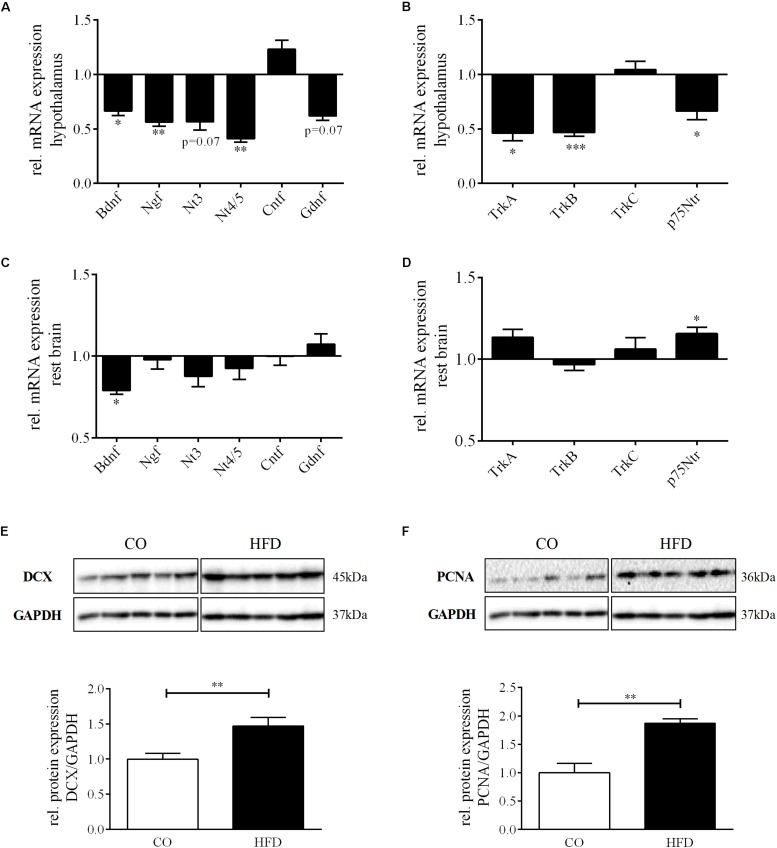
Neurotrophin expression and neurogenesis at P21. Relative gene expression compared to controls (= 1.0) at P21, CO *n* = 8, HFD *n* = 8. **(A)** Hypothalamic neurotrophins Bdnf, Ngf, Nt3, Nt4/5, Cntf, and Gdnf and **(B)** hypothalamic neurotrophin receptors TrkA, TrkB, TrkC, and p75Ntr. **(C)** Rest brain neurotrophins Bdnf, Ngf, Nt3, Nt4/5, Cntf, and Gdnf and **(D)** rest brain neurotrophin receptors TrkA, TrkB, TrkC, and p75Ntr. **(E,F),** Representative Western Blots of DCX and PCNA in hypothalamic tissue at P21. **(E,F)**, Densitometric analysis of protein expression at P21: **(E)** DCX/GAPDH, CO *n* = 5, HFD *n* = 5. **(F)** PCNA/GAPDH, CO *n* = 5, HFD *n* = 5. Data are presented as mean ± SEM. ^∗^*p* < 0.05; ^∗∗^*p* < 0.01; ^∗∗∗^*p* < 0.001; CO, control; HFD, high fat diet.

With regard to the microarray results in the hypothalamus of HFD and CO offspring at P21, we first determined the mRNA expression of several neurotrophic growth factors (*Bdnf*, *Ngf*, *Nt3*, *Nt4/5*, *Cntf*, and *Gndf*) in the hypothalamus of HFD and CO offspring at P21. Interestingly, we found a significant reduction in gene expression of *Bdnf* (66.7% of control; *p* < 0.05), *Ngf* (56.3% of control; *p* < 0.01) and *Nt4/5* (41.0% of control; *p* < 0.01) in HFD offspring compared to controls (Figure 4A). Moreover, there was also a trend toward reduced mRNA expression detectable for Nt3 and Gdnf, with 56.8 and 61.9% of control expression, respectively (*p* = 0.07). In contrast, for Cntf we detected a tendency toward increased gene expression as a result of maternal obesity (*p* = 0.10) (Figure 4A).

To determine whether altered hypothalamic gene expression as a result of maternal obesity is not only found for neurotrophic growth factors but also for their receptors, we quantified gene expression of *TrkA*, *TrkB*, *TrkC*, and *p75Ntr* in the hypothalamus of HFD and CO offspring at P21. We found no change in mRNA expression levels of *TrkC*, but significantly reduced gene expression for *TrkA*, *TrkB*, and *p75Ntr* (Figure 4B).

mRNA expression patterns of both, neurotrophins and neurotrophin receptors, in the rest of the brain did not reflect the pattern that we found in the hypothalamus (Figures 4C,D). However, mRNA expression of *Bdnf* was also found to be significantly reduced (*p* = 0.045) (Figure 4C) and *p75Ntr* significantly increased in the rest of the brain (0.023) (Figure 4D).

### Hypothalamic Neurogenesis and Synaptic Plasticity

To next evaluate the effects of maternal obesity on hypothalamic neuronal network plasticity, we first determined protein expression of doublecortin (DCX), a specific marker for newborn neurons, in the hypothalamus of HFD and CO offspring at P21. Hypothalamic DCX protein expression was significantly increased in HFD offspring compared to controls indicating enhanced hypothalamic neurogenesis (Figure 4E). Supporting this finding, also hypothalamic protein levels of proliferating cell nuclear antigen (PCNA), a marker for DNA synthesis but also DNA repair (Frade and Ovejero-Benito, 2015), was significantly increased in HFD offspring (Figure 4F).

**FIGURE 5:**
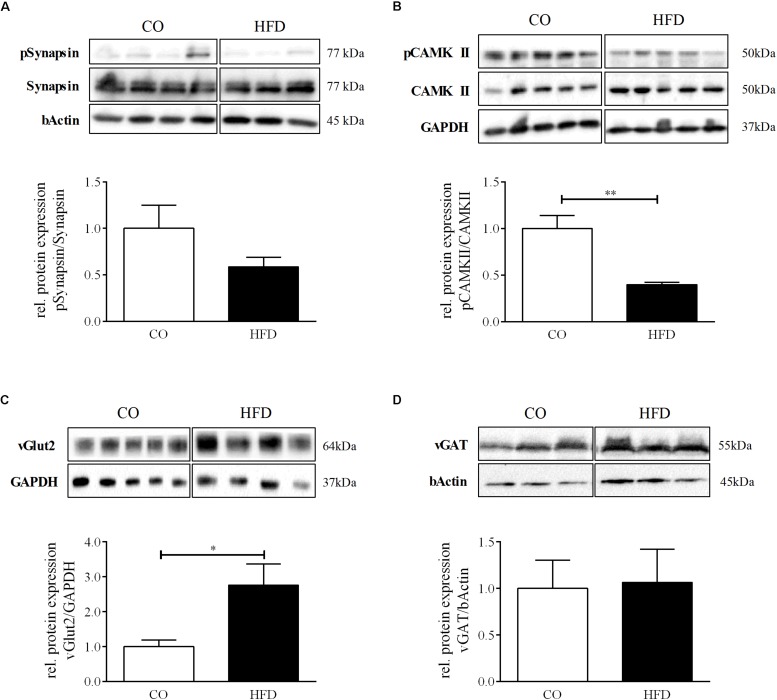
Hypothalamic synaptic plasticity at P21. **(A–D)** Representative Western Blots of pSynapsin, pCAMKII, vGlut2, and vGAT in hypothalamic tissue at P21. **(A–D)** Densitometric analysis of protein expression at P21: **(A)** pSynapsin/Synapsin, CO *n* = 4, HFD *n* = 4. **(B)** pCAMKII/CAMKII, CO *n* = 4, HFD *n* = 4. **(C)** vGLUT2/GAPDH, CO *n* = 5, HFD *n* = 4. **(D)** vGAT/GAPDH, CO *n* = 3, HFD *n* = 3. Data are presented as mean ± SEM. ^∗^*p* < 0.05; CO, control; HFD, high fat diet.

We additionally quantified phosphorylation of synapsin, which is known to facilitate the transport of neurotransmitter-filled vesicles to the synapse during an action potential and thereby promotes synaptic plasticity. There was no statistically significant difference between groups (Figure 5A). However, we detected significantly reduced protein levels of phosphorylated Ca^2+^/calmodulin-dependent protein kinase II (CAMKII) in HFD offspring at P21, indicating changes in hypothalamic synaptic plasticity and efficacy as a result of maternal obesity (Figure 5B). To next evaluate the excitatory and inhibitory synaptic tone in the hypothalamus at P21, we measured hypothalamic protein expression levels of the vesicular glutamate transporter 2 (vGLUT2), a marker for excitatory synapses, and vesicular GABA transporter (vGat), a marker for inhibitory synapses. HFD offspring displayed significantly increased levels of vGLUT2 compared to controls, while vGAT remained unaltered (Figures 5C,D), indicating an overall increased excitatory tone in the hypothalamus of HFD offspring.

### Hypothalamic MAPK Signaling

**FIGURE 6:**
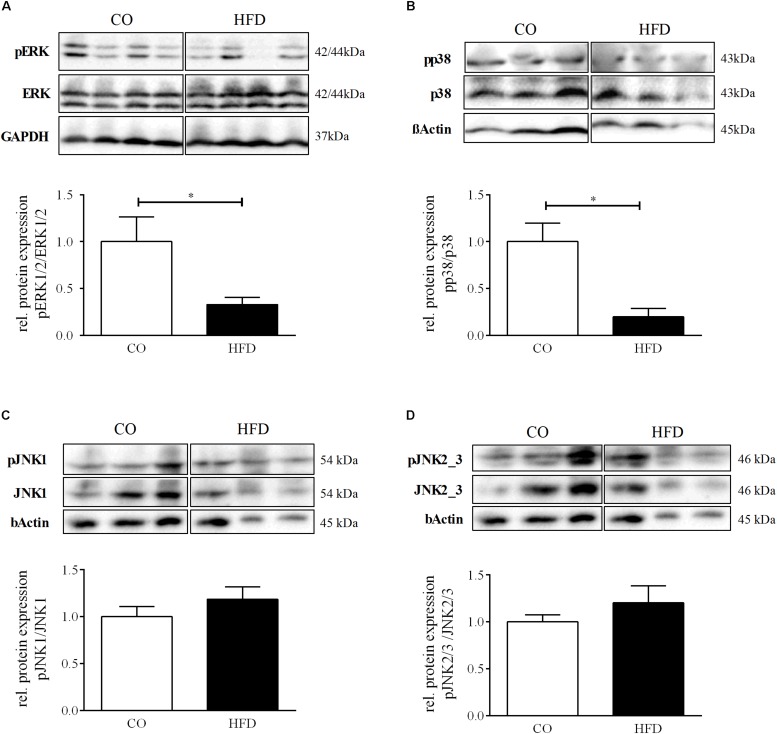
Hypothalamic MAP kinases signaling. **(A–D)** Representative Western Blots of pERK, pp38, pJNK1, pJNK2/3 in hypothalamic tissue at P21. **(A–D)** Densitometric analysis of protein expression at P21: **(A)** pERK/ERK, CO *n* = 4, HFD *n* = 4. **(B)** pp38/p38, CO *n* = 4, HFD *n* = 4. **(C)** pJNK1/JNK1, CO *n* = 4, HFD *n* = 4. **(D)** pJNK2/3/JNK2/3, CO *n* = 4, HFD *n* = 4. Data are presented as mean ± SEM. ^∗^*p* < 0.05; CO, control; HFD, high fat diet.

Neurotrophin-associated signaling has been shown to converge on the levels of mitogen activated protein kinase (MAPK) signaling (Thomas and Huganir, 2004), an intracellular cascade closely tied to synaptic plasticity in several parts of the brain (Borrie et al., 2017). To investigate whether intracellular MAPK signaling in the hypothalamus of the offspring might be affected by maternal obesity, we determined total protein amount and phosphorylation of the MAP kinases extracellular signal regulated kinases 1/2 (ERK1/2), p38, c-Jun N-terminal kinase 1 (JNK1), and JNK2/3 in the hypothalamus of offspring of obese and lean mouse dams on P21. The ratio between phosphorylated and total amount of ERK1/2 was significantly reduced to 32.9% (*p* = 0.041) (Figure 6A) in the hypothalamus of HFD offspring indicating a decreased hypothalamic activation of the ERK1/2 pathway following maternal obesity. Moreover, for p38 we revealed significant reduction of pp38 in relation to total p38 to 19.8% (*p* = 0.002), indicating reduced activation of the p38 pathway in the hypothalamus of HFD offspring (Figure 6B). There were no significant changes in phosphorylation of JNK1 and JNK2/3 in the hypothalamus at P21 (Figures 6C,D).

## Discussion

In the present study, we determined the effects of maternal obesity on the offspring’s hypothalamic glucose sensing and insulin signaling mechanisms, assessed the global gene expression profile of the hypothalamus, and addressed hypothalamic neurotrophin expression and associated mechanisms of synaptic plasticity at P21.

We were able to describe numerous novel hypothalamic changes in HFD offspring: (a) increased IBA1 expression, (b) a global gene expression pattern indicative of major changes in neurogenesis and plasticity, and (c) reduced neurotrophin expression, changes in neurogenesis and plasticity marker expression as well as reduced MAPK signaling (ERK1/2 and p38).

In detail, we showed that maternal obesity resulted in a massive increase in hypothalamic insulin signaling at P21 and we found increased expression of hypothalamic IBA1, a marker for activated microglia. Activated microglia has been shown to promote reactive gliosis in the hypothalamus (Valdearcos et al., 2017), a mechanism directly affecting hypothalamic connectivity (Dorfman and Thaler, 2015). Just recently, acute insulin administration in mice was shown to induce both, hypothalamic AgRP expression and microglia activation (Winkler et al., 2019) suggesting a role for microglial cells in close vicinity to orexigenic neurons in facilitating the release of hunger signals to counteract hypoglycemia. In light of this hypothesis, the strong and long-lasting increase in hypothalamic insulin signaling in HFD offspring at P21 might be one reason for the observed activation of microglia in order to prevent potential hypoglycemia. This would result in increased sensation of hunger and the promotion of reactive hypothalamic gliosis with all its consequences for formation and function of hypothalamic feeding networks (Thaler et al., 2012; Valdearcos et al., 2017). This interpretation is supported by the finding of Vogt et al., who could show that blockade of insulin signaling in hypothalamic anorexigenic POMC neurons protects the offspring from maternal HFD-induced malformation of POMC neuron projections (Vogt et al., 2014). It is important to note that increased hypothalamic insulin signaling at P21 in our model of maternal obesity seems to precede insulin resistance in the brain and peripheral organs in adult offspring as shown by previous work of our group (Schmitz et al., 2018) and others (Samuelsson et al., 2008; Akyol et al., 2012; Gomes et al., 2018; George et al., 2019). Thus, it is tempting to speculate whether adult-onset hypothalamic insulin resistance might be a functional consequence of increased hypothalamic insulin signaling during early life. With regard to leptin’s effect at P21, it is worth mentioning that leptin is known to inhibit AMPK in the arcuate nucleus and reduce food intake (Hardie, 2015). Thus, it seems plausible that increased hypothalamic leptin action at P21 reflects the overfed state in HFD offspring. It would, however, be of great interest to attribute the reduction in phosphorylated hypothalamic AMPK found in our model at P21 to a specific cell type to further understand leptin’s role in hypothalamic AMPK signaling.

Furthermore, we performed microarray analysis of hypothalamic tissue of CO and HFD offspring at P21. These data revealed significantly different gene expression signatures as a result of maternal obesity. Here we present all differentially expressed genes arising from the comparison of CO and HFD offspring (Supplementary Tables 3, 4). Among the most upregulated genes in HFD offspring we found multiple zinc finger proteins. Analyzing the most prominently downregulated genes in HFD offspring, we found amongst others *Map3k10* and *Cntfr*, a member of the MAP kinase family and a neurotrophin receptor. Furthermore, among the affected biological processes by maternal HFD, many GO-Terms referring to neurogenesis, axogenesis, and synaptic plasticity peaked out. By qPCR measurements in hypothalamic tissue, we confirmed that, indeed, HFD offspring displays significantly reduced hypothalamic mRNA expression levels for various neurotrophins and neurotrophin receptors, including *Bdnf* and its receptor *TrkB*. This finding is interesting in several ways. Besides its direct effect on synaptogenesis and neuronal plasticity (Unger et al., 2007), BDNF has been shown to have a clear anorexigenic effect (Molteni et al., 2004; Rask-Andersen et al., 2011). Heterozygous BDNF-knockout mice display hyperphagia with significantly increased body weight and elevated serum leptin and insulin levels along with decreased hypothalamic *Bdnf* mRNA expression (Kernie et al., 2000). Furthermore, intra-cerebroventricular application of BDNF or other TrkB ligands can reverse this effect and cause significant weight loss (Kernie et al., 2000; Burns et al., 2010; Fox et al., 2013), and BDNF injection specifically into the ventromedial nucleus of the hypothalamus leads to reduced food intake and an increase in energy expenditure (Wang et al., 2007). Moreover, also reduced TrkB receptor expression in the brain of mice leads to increased body weight and food intake (Xu et al., 2003). Keeping all that in mind, it is well conceivable that reduced hypothalamic BDNF signal transduction in offspring of obese mouse dams as indicated by reduced gene expression of both, *Bdnf* and TrkB, might contribute to their predisposition for obesity on P21 and permanently shape hypothalamic feeding networks to metabolic dysfunction. Future studies are needed to prove this hypothesis.

Information on the role of other members of the neurotrophin family and their receptors in energy homeostasis regulation is scarce. However, it is known that intra-cerebroventricular injection of NGF or NT4/5 also causes weight loss in mice (Kernie et al., 2000). NGF acts mainly through the TrkA receptor, whereas NT4/5 has been shown to activate TrkB receptor signaling (Glebova and Ginty, 2005). Thus, the hypothalamic gene expression pattern in the offspring of obese mouse dams in our experiment suggests an overall reduction in hypothalamic TrkA and B signaling following maternal obesity. This could contribute to the significantly reduced activation of the receptor associated MAP kinases ERK1/2 and p38 (Chaves et al., 2013; Wuhanqimuge et al., 2013). Both, ERK1/2 and p38, are sensitive to feeding status and show increased activation upon fasting in the hypothalamus of mice (Ueyama et al., 2004). At the same time, ERK1/2 signaling plays an important role in synapse formation (Huang and Reichardt, 2001; Alonso et al., 2004; Hans et al., 2004) and participates in long-term synaptic plasticity in hippocampus and sensory neurons (Martin et al., 1997). Various studies indicate that MAPKs are located in synaptic terminals influencing short- and long-term plasticity by phosphorylation of synaptic targets such as synapsin (Jovanovic et al., 2000; Sweatt, 2004; Boggio et al., 2007; Giachello et al., 2010). Thus, a reduction in Trk-mediated MAPK activation along with a reduction in synapsin phosphorylation to approximately 50% following maternal obesity (although not statistically significant) would suggest a link between maternal body weight and both, the offspring’s hypothalamic neuronal plasticity and their metabolic phenotype on P21. However, it is important to note that we did only observe a trend toward reduced synapsin phosphorylation in this study.

In addition to the above mentioned MAPK signaling pathways, neurotrophins also activate the phospholipase C gamma (PLCgamma) and the phosphatidylinositol 3-kinase (PI3K) pathways. While we did not address the role of PLCgamma signaling in this study, we determined hypothalamic AKT phosphorylation, an important downstream messenger of PI3K in HFD and CO offspring at P21. In contrast to the reduction in MAPK signaling, we found a massive increase in AKT phosphorylation in the hypothalamus of HFD offspring accompanied by increased phosphorylation of the downstream target GSK3ß. This finding cannot be explained by the observed changes in neurotrophin expression. As mentioned above, however, we found a strong increase in hypothalamic insulin signaling in HFD offspring. Thus, one could speculate that a potential effect of altered neurotrophin signaling is masked by the strong insulin action on hypothalamic cells at this time point. Being aware of the fact that reduced BDNF and NGF serum and tissue levels have been associated with hyperinsulinemic states like obesity and type-2-diabetes (Molteni et al., 2002; Geroldi et al., 2006; Suwa et al., 2006; Bullo et al., 2007; Krabbe et al., 2007; Fujinami et al., 2008; Yamanaka et al., 2008; Golden et al., 2010), even a causal relationship between overactive insulin signaling and decreased neurotrophin levels as part of a negative feedback mechanism is imaginable (Ding et al., 2006). Of course, further experiments will be needed to prove this hypothesis including deeper analysis of functional insulin sensitivity at P21.

The overlap in effects of neurotrophins and other metabolic cues on MAPKs and PI3K might also explain the effects of maternal obesity on expression of markers of synaptic plasticity in the hypothalamus of the offspring. Phosphorylation of synapsin in the hypothalamus shows a tendency toward reduction following maternal obesity along with significantly reduced pCAMKII protein levels. CAMKII is a protein involved in synaptic plasticity, memory and learning and its phosphorylated form is considered constitutively active. CAMKII activity is required for induction of long-term potentiation in the hippocampus and the associated structural plasticity of dendritic spines (Murakoshi et al., 2017). To our knowledge, CAMKII has so far not been assessed in the hypothalamus with regard to network formation of feeding circuits. However, reports on leptin modulating CAMKII activity in the hippocampus *in vitro* in a dose-dependent manner (Vogt et al., 2014) and altered hypothalamic CAMKII activity in diabetic rats (Ramakrishnan et al., 2005b) support our hypothesis that hormones or other metabolic factors might affect hypothalamic spine formation and synaptic activity via CAMKII-mediated processes. Interestingly, reduced hypothalamic CAMKII activity *in vitro* goes along with reduced p38 levels (Ramakrishnan et al., 2005a). And also, ERK1/2 has been functionally linked to synapsin expression *in vitro* (Chen et al., 2018). Both findings might suggest a possible direct connection between reduced MAP kinase signaling and the reduction of expression of both plasticity markers found in the hypothalamus of HFD offspring.

To investigate the overall synaptic tone in the hypothalamus, we set out to quantify vGLUT2 and vGAT protein expression representing excitatory and inhibitory synapses, respectively. While hypothalamic vGAT protein expression did not differ between groups, hypothalamic vGLUT2 protein levels were significantly increased in HFD offspring, indicating an overall increase in excitatory synapses. The ratio of excitatory and inhibitory synaptic inputs to hypothalamic feeding neurons is known to change in response to metabolic hormones, such as leptin and ghrelin (He et al., 2018). Within the hypothalamus, vGlut2 expressing neurons are most abundant in the ventromedial hypothalamic nucleus (VMH) (Tong et al., 2007). Interestingly, mice lacking vGlut2 in the VMH lack the ability to counteract hypoglycemia caused by insulin administration (Tong et al., 2007). Thus, elevated hypothalamic vGLUT2 expression in HFD offspring might directly result from sustained insulin signaling as described before. However, only recently, vGlut2 neurons in the nucleus arcuatus were found to be fast-acting anorexigenic neuronal populations that interconnect peripheral signals like CCK or leptin with other ARC neurons or directly project to the PVN (Guillebaud, 2019). Hypothalamic vGlut2 neurons were shown to be leptin sensitive (Guillebaud, 2019). Thus, increased leptin serum levels, increased hypothalamic leptin receptor protein expression and increased hypothalamic vGLUT2 expression in HFD offspring of our model confirm this recently described functional relationship. As leptin and vGlut2 are both involved in promoting satiety, upregulation of vGlut2 might be a compensatory mechanism of HFD offspring to counteract elevated body weight and fat pad weight at P21. Only future experiments that address the localization of vGlut2 within the different nuclear regions of the hypothalamus can answer the question which of these two conflicting interpretations is adequate or which effect might outweigh the other.

Interestingly, and in contrast to the impression of reduced hypothalamic plasticity marker expression in HFD offspring at P21, hypothalamic markers for proliferation and neurogenesis (DCX and PCNA) were significantly increased. This is in accordance with the only other study that addressed hypothalamic neurogenesis in the context of maternal HFD feeding so far (Chang et al., 2008). Chang et al. elegantly assessed neurogenesis in different hypothalamic nuclei at several time points during lactation and revealed that the effects on neurogenesis differ significantly between the distinct nuclei, with the most prominent increase in neurogenesis in the periventricular nucleus and the lateral hypothalamus. However, rat dams in their study received HFD only during part of the gestation and did not display an obese phenotype. In contrast, offspring exposure to gestational diabetes was shown to result in the malformation of medio-basal hypothalamic nuclei, which was suggested to be secondary to reduced neuron formation (Harder et al., 2001; Fahrenkrog et al., 2004; Franke et al., 2005; Dearden and Ozanne, 2015). Neurogenesis is generally thought to have a positive effect on network plasticity. However, recent studies reveal that in the hypothalamus, neuronal precursor cells are capable of differentiating into orexigenic or anorexigenic neurons in response to nutritional signals. Hypothalamic neurogenesis may thus act as an adaptive mechanism in order to respond to changes in food supply (Recabal et al., 2017). So, together with insulin’s postulated effect on IBA1 and vGlut2 expression in our model, increased neurogenesis (of orexigenic neurons?) in the hypothalamus of HFD offspring might thus also be interpreted as part of the physiologic response to prevent of hypoglycemia. Co-localization of DCX and orexigenic and anorexigenic cell markers would be helpful to test this hypothesis and evaluate whether, indeed, the HFD offspring’s neuronal precursor cells are more prone to differentiate into orexigenic neurons.

During pregnancy and lactation, the offspring of obese mothers is inevitably exposed to a variety of altered circulating hormonal and nutritional signals. Especially, the quality of ingested fatty acids seems to be of important matter for brain development and function of the offspring, as ingestion of increased amounts of saturated or trans fatty acids by the mother even in the absence of obesity has been shown to cause hypothalamic dysfunction in mice and rat offspring (Pimentel et al., 2011; Rother et al., 2012). We found reduced hypothalamic neurotrophin signaling following maternal obesity that was induced by ingestion of a diet rich in saturated fatty acids. In a very similar animal model, maternal obesity has been shown to cause BDNF deficiency in the hippocampus and deficits in spatial learning in the offspring (Tozuka et al., 2010). Also, omega-3-fatty acid deficiency during pregnancy and lactation has been shown to alter BDNF-TrkB-signaling in the hypothalamus and cause anxiety-like behavior in the rat (Bhatia et al., 2011). Taken together, hypothalamic neurotrophin signaling might be influenced by the quality and quantity of dietary fatty acids ingested by the dam. This hypothesis is further supported by the fact that also in adult rats, high-fat feeding causes reduced BDNF-signaling in the hippocampus, that can be restored upon reduced dietary fatty acid intake (Woo et al., 2013).

The timeliness of linking diet-induced changes in hypothalamic neurotrophin signaling to alterations in energy homeostasis is further substantiated by a report of Byerly et al. (2013) who identified neuron-derived neurotrophic factor (NENF) as a novel secreted protein in the hypothalamus regulating appetite by interacting with melanocortin signaling. Thus, neurotrophin action in the hypothalamus is gaining more and more attention as an important modulator of hypothalamic control of energy balance – either by directly modulating synaptic network formation and function or by interacting with established pathways of energy homeostasis regulation like the melanocortin system.

We aimed to shed light on the molecular mechanisms responsible for hypothalamic energy balance regulation from a completely new perspective. There are several limitations to our study – including the fact that we were not able to attribute the observed effects to specific nuclear regions of the hypothalamus and that we are lacking a direct mechanistic link between our observations and hypothalamic network formation in our animals. Yet, taken together, there is clear evidence that body weight and feeding status of the mother has an impact on the offspring’s hypothalamic neurotrophin signaling and expression of proteins involved in synaptogenesis and neurotransmitter release. Future experiments will have to show whether the observed changes are the underlying mechanism for altered wiring of hypothalamic feeding circuits and might therefore explain the predisposition for disturbed energy homeostasis as a consequence of maternal obesity. Another limitation of the study is that only male offspring were analyzed in the animal model. Some effects of developmental programming on long-term offspring health are gender-specific (Howie et al., 2009; Carter et al., 2012; de Souza et al., 2015). De Souza et al. who recently published studies examining the effects of maternal obesity on the offspring, used only male offspring. Our data is comparable with those studies (Alfaradhi et al., 2016; Frihauf et al., 2016; Liang et al., 2016; Beeson et al., 2018; Berends et al., 2018; Loche et al., 2018).

## Conclusion

Taken together, we detected a global gene expression pattern in HFD offspring at P21 that was indicative of major changes in hypothalamic neurogenesis and plasticity. Targeted analysis of hypothalamic neurotrophin expression, neurogenesis and plasticity marker expression, and MAPK signaling (ERK1/2 and p38) confirmed our hypothesis that hypothalamic neurotrophin associated MAPK signaling might contribute to the pathogenesis of the offspring’s hypothalamic feeding network dysfunction in maternal obesity. Thereby, we hope to add a puzzle piece to the ongoing search for targets for the prevention and treatment of adult metabolic diseases.

## Data Availability

The data discussed in this publication have been deposited in NCBI’s Gene Expression Omnibus (Edgar et al., 2002) and are accessible through GEO Series accession number GSE135830^3^. The raw data supporting the conclusions of this manuscript will be made available by the authors, without undue reservation, to any qualified researcher. Additionally, the raw data is included in the Supplementary Files.

## Ethics Statement

The animal study was reviewed and approved by the Landesamt für Natur, Umwelt und Verbraucherschutz Nordrhein-Westfalen (Permit Number: 37.09.292).

## Author Contributions

Each author has made an important scientific contribution to the study. IB-G, RJ, JD, and EH-R participated in the study design. IB-G and RJ carried out the animal experiments. SB, C-SK, LB, RJ, IB-G, and CV performed the molecular analyses. IB-G, RJ, and EH-R analyzed and interpreted the data. AO performed the microarray analysis. LS, NF, SB, TH, SA, and JD carefully revised the manuscript. EH-R was the guarantor of this work.

## Conflict of Interest Statement

The authors declare that the research was conducted in the absence of any commercial or financial relationships that could be construed as a potential conflict of interest.

## Footnotes

1 https://david-d.ncifcrf.gov/

2 https://www.ncbi.nlm.nih.gov/geo/query/acc.cgi?acc=GSE135830

3 https://www.ncbi.nlm.nih.gov/geo/query/acc.cgi?acc=GSE135830

## Abbreviations

(p)AKT: (phosphorylated) RAC-alpha serine/threonine-protein kinase
(p)AMPK: (phosphorylated) 5 ′ -adenosine monophosphate-activated protein kinase
(p)CAMKII: (phosphorylated) Ca^2+^/calmodulin-dependent protein kinase II
(p)ERK: (phosphorylated) extracellular-signal regulated kinases
(p)GSK3beta: (phosphorylated) glycogen synthase kinase 3 beta
(p)JNK1: (phosphorylated) c-JunN-terminal kinase 1
(p)JNK2: (phosphorylated) c-JunN-terminal kinase 2
(p)JNK3: (phosphorylated) c-JunN-terminal kinase 3
(p)p38: (phosphorylated) p38 mitogen-activated protein kinase
ARC: arcuate nucleus of the hypothalamus
BDNF: brain-derived neurotrophic factor
CNTF: ciliary neurotrophic factor
CO: control group
DCX: doublecortin
G: gestational day
GAPDH: glycerinaldehyd-3-phosphat-dehydrogenase
GDNF: glial cell derived neurotrophic factor
GO-terms: gene ontology terms
GTT: glucose tolerance test
HFD: high fat diet
HRP: horseradish peroxidase
IBA1: ionized calcium-binding adapter molecule 1
InsR: insulin receptor
KEGG: Kyoto Encyclopedia of Genes and Genomes
LepR: leptin receptor
MAPK: mitogen activated protein kinase
NENF: neuron-derived neurotrophic factor
NGF: nerve growth factor
NPY: neuropeptide Y
NT3: neurotrophin 3
NT4/5: neurotrophin 4/5
P: postnatal day
p75NTR: p75 neurotrophin receptor
PCLgamma: phospholipase C gamma
PCNA: proliferating cell nuclear antigen
PI3K: phosphatidylinositol 3 kinase
POMC: propriomelanocortin
PVN: paraventricular nucleus
TrkA: tropomyosin receptor kinase A
TrkB: tropomyosin receptor kinase B
TrkC: Tropomyosin receptor kinase C
vGAT: vesicular gamma-aminobutyric acid (GABA) transportervGlut2 Vesicular glutamate transporter 2.
